# Lower diurnal HPA-axis activity in male hypertensive and coronary heart disease patients predicts future CHD risk

**DOI:** 10.3389/fendo.2023.1080938

**Published:** 2023-03-10

**Authors:** Cathy Degroote, Roland von Känel, Livia Thomas, Claudia Zuccarella-Hackl, Nadine Messerli-Bürgy, Hugo Saner, Roland Wiest, Petra H. Wirtz

**Affiliations:** ^1^ Biological Work and Health Psychology, University of Konstanz, Konstanz, Germany; ^2^ Department of Consultation-Liaison Psychiatry and Psychosomatic Medicine, University Hospital Zurich, University of Zurich, Zurich, Switzerland; ^3^ Department of Psychology, University of Bern, Bern, Switzerland; ^4^ Institute of Psychology, University of Lausanne, Lausanne, Switzerland; ^5^ Institute for Social and Preventive Medicine, University of Bern, Bern, Switzerland; ^6^ Support Center of Advanced Neuroimaging, Institute of Diagnostic and Interventional Neuroradiology, University Hospital Bern, University of Bern, Bern, Switzerland; ^7^ Centre for the Advanced Study of Collective Behaviour, University of Konstanz, Konstanz, Germany

**Keywords:** HPA-axis, cortisol, coronary heart disease, hypertension, coagulation

## Abstract

**Background:**

Coronary heart disease (CHD) and its major risk factor hypertension have both been associated with altered activity of the hypothalamus-pituitary-adrenal (HPA)-axis but the biological mechanisms underlying prospective associations with adverse disease outcomes are unclear. We investigated diurnal HPA-axis activity in CHD-patients, hypertensive (HT) and healthy normotensive men (NT) and tested for prospective associations with biological CHD risk factors.

**Methods:**

Eighty-three male CHD-patients, 54 HT and 54 NT men repeatedly measured salivary cortisol over two consecutive days. Prospective CHD risk was assessed by changes between baseline and follow-up in the prothrombotic factors D-dimer and fibrinogen, the pro-inflammatory measures interleukin (IL)-6, tumor necrosis factor-alpha (TNF-α), and acute phase protein C-reactive protein (CRP), as well as blood lipids in terms of total cholesterol (tChol)/high-density-lipoprotein cholesterol (HDL)-ratio. We aggregated coagulation and inflammatory measures to respective indices.

**Results:**

The groups differed in repeated daytime cortisol (dayCort) secretion (*p*=.005,η^2^_p_=.03,*f*=0.18) and cortisol awakening response (CAR) (*p*=.006,η^2^_p_=.03,*f*=0.18), with similarly lower overall dayCort and CAR in CHD-patients and HT, as compared to NT. The groups differed further in cortisol at awakening (*p*=.015,η^2^_p_=.04,*f*=0.20) with highest levels in HT (*p*´s≤.050), and in diurnal slope between waking and evening cortisol (*p*=.033,η^2^_p_=.04,*f*=0.20) with steepest slopes in HT (*p*´s≤.039), although in part not independent of confounders. Lower aggregated dayCort and CAR in terms of area-under-the-curve (AUC) independently predicted increases in future overall CHD risk (AUC_dayCort_: *p*=.021,η^2^_p_=.10,*f*=0.33;AUC_CAR_: *p*=.028,η^2^_p_=.09,*f*=0.31) 3.00 ± 0.06(*SEM*) years later, with risk prediction most pronounced in fibrinogen (AUC_dayCort_: *p*=.017,Δ*R* ^2^= 0.12;AUC_CAR_: *p*=.082).

**Conclusion:**

We found evidence for an HPA-axis hypoactivity in CHD and HT with lower diurnal HPA-axis activity predicting increases in cardiovascular risk as evidenced by increases in circulating levels of biomarkers of atherothrombotic risk. Down-regulation of basal HPA-axis activity may contribute to the pathogenesis of atherosclerosis and thrombosis in CHD *via* effects on coagulation.

## 1 Introduction

Coronary heart disease (CHD) ranks among the leading causes of death in western countries (1). A major risk factor for CHD is hypertension (HT), a chronic elevation of blood pressure (BP) (2). Most individuals with hypertension are diagnosed with “essential hypertension” with unknown medical cause (3, 4). HT and CHD have both been associated with alterations in diurnal activity of the hypothalamus-pituitary-adrenal (HPA)-axis (see below e.g. (5, 6)), but its relevance with respect to mechanisms of disease progression is not fully understood.

The HPA-axis end-product cortisol is important for the integrity of central nervous system function and for maintenance of cardiovascular, metabolic, and immune homeostasis (7). The basal HPA-axis activity follows a diurnal rhythm characterized by the cortisol awakening response (CAR), a sharp rise by a about 50% to over 100% within the first 30-to-45min after awakening (8–11). This morning peak is followed by a circadian decline with a steady decrease of cortisol throughout the day and minimum levels at midnight (12, 13). Dysregulated circadian cortisol profiles often comprise either high cortisol levels throughout the day and a flattened diurnal rhythm (HPA-axis hyperactivity), or lower overall cortisol secretion with a flatter diurnal slope and lower morning cortisol levels (HPA-axis hypoactivity) [for review see (14)].

A variety of studies *cross-sectionally* assessed basal HPA-axis activity in HT and heart patients or individuals with CHD-symptoms, respectively. Basal HPA-axis activity was compared between individuals with *HT* and normotensives (i.e., a normal blood pressure, NT). Male and female HT who discontinued medication-intake had higher morning cortisol levels compared to NT (15), but not unequivocally (6). With respect to the CAR, we previously found a blunted CAR in unmedicated HT (6) and also medicated hypertensive men and women showed lower aggregated total CAR compared to NT (16). Similarly, in predominantly non-hypertensive male and female participants, higher BP related to a lower aggregated total CAR (17). Regarding diurnal cortisol profiles, we could not previously observe differences between medication-free HT and NT (6). Evening cortisol was higher in HT compared to NT (15). In *heart disease patients*, salivary waking or morning cortisol was not associated with CHD (or markers of subclinical CHD) in most studies (18–20). Patients with cardiovascular disease (CVD) showed flatter CARs than non-CVD participants (21) and hypertensive patients with acute coronary syndrome (ACS) had smaller aggregated CARs than normotensive patients (5), but there are also contradicting findings (18). With respect to salivary cortisol daytime levels, CHD-patients did not differ in cortisol slopes or total cortisol output over the day from participants without a CHD-diagnosis but symptoms (18). Regarding *associations with markers of subclinical CHD*, salivary waking or morning cortisol was not associated with CHD (or markers of subclinical CHD) in most studies (18–20). Various measures reflecting a flatter CAR were associated with higher values of intima-media-thickness (22) and ankle-brachial-index (ABI) (19) in women and coronary artery calcification (CAC) in men and women (19), but not unequivocally [see also (23)]. With respect to diurnal cortisol, a flatter slope over the day was associated with the recurrence of cardiac symptoms in ACS-patients (5) and with higher CAC in population-based studies (24). Also, higher total cortisol output was related to more carotid plaques (25). However, there are also diverging diurnal cortisol findings (19, 23–25). Bedtime cortisol levels were higher in ACS-patients compared to healthy controls (20). Taken together, the above-described cross-sectional findings point to a reduced CAR, flatter slopes over the day, and higher cortisol evening levels in hypertensive individuals as well as in heart patients. However, to the best of our knowledge, it has not yet been investigated whether HT differ from CHD-patients in their basal HPA-axis activity or whether the separately observed dysregulations are comparable in both groups.

*Prospective evidence* is emerging that a dysregulated basal HPA-axis activity relates to adverse heart disease outcomes, but the underlying biological mechanisms with respect to disease progression are unclear. Lower waking cortisol in patients undergoing coronary artery bypass graft (CABG) surgery predicted cardiac events and death about 3 years later (26). Moreover, a flatter diurnal cortisol decline predicted cardiac events and death in CABG-patients (26), and CVD-related mortality in Whitehall-II participants (27). Finally, higher bedtime or evening cortisol levels predicted mortality risk (27, 28), adverse clinical outcomes (26), and incidence of fatal CHD (29), predominantly in heart patients. The mechanisms underlying these prospective associations have rarely been investigated. So far, only two non-patient studies suggest prospective associations that point to the process of atherosclerosis. In policemen, a flatter aggregated CAR predicted a larger 7-year mean decline in brachial artery flow-mediated dilation indicative of endothelial dysfunction (30). Moreover, in a population-based study, healthy women with flatter diurnal cortisol slopes and higher bedtime levels showed greater progression of aortic stiffness 5 years later (31).

Independent biological CHD risk factors that underly the process of atherosclerosis and thrombosis include markers of coagulation, inflammation, and hyperlipidemia (32–35). To date, despite evidence for cross-sectional associations (20, 36–39), prospective evidence addressing associations between basal HPA-axis activity and independent biological CHD risk factors is lacking so far, not only in healthy participants, but also in hypertension or CHD. A better understanding of the biological mechanisms underlying disease progression may have implications for longer-term therapy in vulnerable populations such as HT and heart patients.

To close the above described gaps in current knowledge, the first objective of our study was to cross-sectionally compare diurnal HPA-axis activity between CHD-patients, HT, and controls with neither HT or CHD (NT). We repeatedly measured salivary cortisol over two consecutive days and hypothesized a blunted CAR, flatter diurnal slopes, and higher evening cortisol levels in both, CHD-patients and HT, as compared to NT. Second, to obtain new mechanistic insights with respect to the clinical relevance of basal HPA-axis activity, we prospectively investigated whether cortisol would predict changes in biological CHD risk factors including markers of coagulation, inflammation, and hyperlipidemia over a mean follow-up of 3 years.

## 2 Materials and methods

### 2.1 Study participants

The current investigation is part of a study program assessing psychoneurobiological mechanisms in essential hypertension and CHD (40–43). It was approved by the ethics committee of the Canton of Bern, Switzerland and the study protocol is in accordance with the Declaration of Helsinki. All participants provided written informed consent and were financially compensated for each assessment with CHF 20.

We restricted our study sample to male individuals given the prevalence of cardiovascular diseases at earlier age (e.g. (44, 45)), given sex differences in HPA-axis activity (e.g. (10)), and given the differences between men and women regarding the associations of diurnal cortisol secretion and CHD-symptoms (e.g. (22)). We recruited male participants with either a diagnosis of CHD, apparently healthy individuals with essential hypertension without CHD, or healthy normotensive controls and asked them to provide saliva samples for the assessment of diurnal cortisol profiles. The final cross-sectional sample comprised 191 participants, with 83 CHD-patients, 54 HT (42 medication-free, 12 medicated) as well as 54 NT. All study participants were invited for a follow-up assessment, with 106 subjects (NT:*n*=32, HT:*n*=31, CHD-patients:*n*=43) completing both assessments (after an average of 3yrs (3.00 ± 0.06 *SEM*). More information is detailed in the **Supplemental Material**.

Reasons for drop-out at follow-up included could not be reached by phone (*n*=9), lack of time (*n*=16), no interest (*n*=12), excessive demands (*n*=7), severe illness (*n*=8), meanwhile living abroad (*n*=2), discontent with the study management (*n*=1), deceased (*n*=2), or no specific reason given (*n*=26). Further, two participants had to be excluded because of acute infection on the follow-up study day. Notably, due to organizational reasons (relocation of the working group from Bern to Konstanz) the number of available follow-up time-slots per month was substantially reduced compared to the baseline assessment, resulting in a prolonged follow-up time and potentially responsible for the attrition. Attrition was comparable across the study groups (drop-out rate: NT=40.7%; HT=42.6%; CHD=48.2%; Chi^2^(2)=.85, *p*=.66).

#### 2.1.1 Recruitment and general inclusion criteria

*CHD-patients.* We included patients with stable CHD who had been discharged from the Cardiac Prevention and Rehabilitation Clinic of the Bern University Hospital at least 6 months ago. We asked those patients of the Cardiac Prevention and Rehabilitation Clinic of the Bern University Hospital who volunteered to be contacted for the purpose of scientific studies. All patients were diagnosed with CHD based on coronary angiography and we provide information regarding myocardial infarction (MI), left ventricular ejection fraction (LVEF) ≤ 40%, and coronary artery bypass graft surgery (CABG) in **Table 1**. All patients were under medication based on current guidelines and in stable compensated cardiac conditions (46).

**Table 1:** Group characteristics, diurnal cortisol, and intermediate biological CHD risk factors at baseline.

	CHD *n*=83	HT *n*=54	NT *n*=54	*p*			
				NT, HT, vs. CHD	NT vs. HT	NT vs. CHD	HT vs. CHD
Age [years]	65.02 ± 0.99 (44–85)	52.74 ± 1.57 (21–74)	50.80 ± 1.63 (25–78)	**<.001**	.39	**<.001**	**<.001**
BMI [kg/m^2^]	27.85 ± 0.43 (21.97–46.44)	28.51 ± 0.52 (20.35–38.86)	25.27 ± 0.33 (19.78–30.85)	**<.001**	**<.001**	**<.001**	.31
Study BP [mmHg]							
Study SBP	141.57 ± 1.64 (111.67–187.33)	151.96 ± 1.86 (120.67–189.67)	127.09 ± 1.17 (109.33–139.67)	**<.001**	**<.001**	**<.001**	**<.001**
Study DBP	81.20 ± 1.09 (62.33–105.00)	93.99 ± 1.31 (72.67–115.00)	78.28 ± 1.03 (58.33–89.50)	**<.001**	**<.001**	.07	**<.001**
Study MAP	101.33 ± 1.11 (81.89–132.00)	113.31 ± 1.41 (88.67–139.89)	94.55 ± 1.01 (75.33–104.83)	**<.001**	**<.001**	**<.001**	**<.001**
Home BP [mmHg]							
Home SBP		143.52 ± 1.28 (119.17–162.33) *n*=52	122.76 ± 0.86 (105.17–134.60)		**<.001**		
Home DBP		85.67 ± 1.02 (68.33–103.00) *n*=52	72.23 ± 0.80 (60.00–82.33)		**<.001**		
Medication*	*n*=83	*n*=12					
LVEF ≤ 40% [%]	11 (13.3) *n*=81	–	–				
MI [%]	47 (56.6) *n*=81	–	–				
CABG [%]	23 (27.7) *n*=81	–	–				
Smoking [%]	5 (6.0)	–	–				
HbA1c [mmol/mol]	41.49 ± 0.77 (33–72) *n*=80	36.74 ± 0.52 (26–43) *n*=53	36.29 ± 0.49 (26–42) *n*=51	**<.001**	.58	**<.001**	**<.001**
Creatinine [μmol/L]		80.76 ± 1.41 (64–103)					
Sodium [mmol/L]		140.32 ± 0.30 (135–145) *n*=47					
Calcium [mmol/L]		2.37 ± 0.01 (2.11–2.58) *n*=46					
Potassium [mmol/L]		4.12 ± 0.04 (3.70–4.90) *n*=47					
Cortisol							
Awakening [nmol/L]	4.41 ± 0.29 (0.35–18.00)	5.38 ± 0.41 (0.68–14.83)	3.94 ± 0.39 (0.02–15.34)	**.015**	**.006**	.18	.05
16:00h [nmol/L]	1.82 ± 0.14 (0.06–7.38)	1.77 ± 0.12 (0.14–4.29)	1.8 ± 0.16 (0.31–5.15)	.87	.97	.65	.68
22:00h [nmol/L]	0.85 ± 0.07 (0.09–3.14)	0.75 ± 0.12 (0.04–6.10)	0.82 ± 0.10 (0.10–3.77)	.23	.49	.35	.08
Slope_Awake_	-0.23 ± 0.02 (-1.00–0.18)	-0.29 ± 0.03 (-0.87–0.17)	-0.20 ± 0.03 (-0.96–0.17)	**.033**	**.021**	.44	**.039**
Slope_Peak_	-0.46 ± 0.03 (-1.13–0.18)	-0.50 ± 0.03 (-1.11–0.18)	-0.49 ± 0.05 (-1.63–0.04)	.66	.89	.52	.37
*M* wake-up time [h]	6:16 ± 0:05 (3:55–8:00)	6:05 ± 0:06 (4:20–7:27)	5:55 ± 0:06 (3:27–7:07)	**.040**	.27	**.014**	.18
* M* sleep duration [h]	7.51 ± 0.10 (4.92–9.95)	7.28 ± 0.09 (5.94–9.00)	7.04 ± 0.10 (4.94–8.92)	**.004**	.07	**.002**	.15
Coagulation							
Fibrinogen [g/L]	2.85 ± 0.06 (1.65–4.46)	2.62 ± 0.06 (1.47–3.72)	2.6 ± 0.08 (1.57–4.25)	**.010**	.73	**.009**	**.013**
D-dimer [µg/L]	622.64 ± 92.37 (45–5177)	474.04 ± 30.41 (155–1047)	513.46 ± 47.36 (45–1616)	.94	.89	.85	.74
Inflammation							
IL-6 [pg/mL]	0.56 ± 0.04 (0.03–1.57) *n*=82	0.57 ± 0.04 (0.16–1.48) *n*=53	0.50 ± 0.07 (0.22–3.61) *n*=53	.18	**.047**	.19	.54
TNF-α [pg/mL]	2.09 ± 0.09 (0.71–5.11) *n*=82	1.84 ± 0.06 (0.72–3.28) *n*=53	2.07± 0.10 (0.88–4.91) *n*=53	.16	.07	.95	.09
CRP [μg/mL]	2.15 ± 0.22 (0.07–11.55) *n*=78	2.83 ± 0.26 (0.64–8.65) *n*=50	2.06 ± 0.30 (0.11–9.59) *n*=40	**<.004**	**<.001**	.63	**<.002**
tChol/HDL	3.09 ± 0.08 (1.73–5.86) *n*=80	3.99 ± 0.13 (2.38–6.41)	3.66 ± 0.13 (2.03–5.72) *n*=51	**<.001**	.06	**<.001**	**<.001**

Values are *M ± SEM*. CHD, CHD-patients; HT, hypertensive individuals; NT, normotensive individuals; BMI, body mass index; CABG, coronary artery bypass graft surgery; DBP, diastolic blood pressure; LVEF, left ventricular ejection fraction; MAP, mean arterial blood pressure; MI, myocardial infarction; SBP, systolic blood pressure; Slope_Awake_, slope anchored to awakening; Slope_Peak_, slope anchored to peak, *See **Supplemental Table S1**. Statistically significant results are highlighted in bold.

*Essential hypertension and normotension.* We recruited apparently healthy, nonsmoking hypertensive and normotensive men of comparable age by aid of the Swiss-Red-Cross of Bern. Members of our study team accompanied the mobile blood-donation unit that routinely records BP ranges before blood donation. Interested blood donors were given written study information asking for the following inclusion criteria: age between 18-80 years; BP either in the hypertensive or in the normotensive range (see below); smoking less than 5 cigarettes per day; and no alcohol or illicit drug abuse. We accepted intake of antihypertensive medication in a small proportion to increase sample size of hypertensive individuals. Apart from hypertension-related criteria, NT and HT were required to meet the same inclusion and exclusion criteria (alcohol and illicit drug abuse, strenuous exercise, liver and renal diseases, chronic obstructive pulmonary disease, allergies and atopic diathesis, rheumatic diseases, human immunodeficiency virus, cancer, major psychiatric disorders, neurological diseases, and current infectious diseases) as verified by telephone interview using an extensive health questionnaire (42, 43). Four eligible participants (NT:*n*=2, HT:*n*=2) who reported regular medication intake stopped medication one week prior to participating in the study. To exclude potential cases with secondary hypertension, eligible HT provided blood samples for the routine assessment of serum creatinine, calcium, sodium, and potassium (47). No eligible HT was diagnosed with secondary hypertension. We measured HbA1c in all participants. Furthermore, we recruited 12 participants previously diagnosed as hypertensives, who were under antihypertensive medication.

#### 2.1.2 Classification of essential hypertension and normotension

Classification of essential hypertension and normotension of the unmedicated participants was based on a two-step assessment procedure, while medicated hypertensive individuals and CHD-patients were assigned *a priori* to the study groups.

*(1) Home blood pressure measurement.* Following written instructions, interested blood donors were asked to measure BP on three days at home using an upper arm digital blood pressure monitor (Omron M6; Omron-Healthcare-Europe B.V., Hoofdorp, Netherlands). Home BP was to be measured twice a day (once in the morning and in the evening) in a seated position after a 15-minute rest. Participants were recruited as hypertensive if the average home systolic BP (SBP) was ≥135mmHg and/or the average home diastolic BP (DBP) was ≥85mmHg according to recommendations for home BP measurements (48). Correspondingly, participants were recruited as normotensive if the average home SBP was <135mmHg and the average home DBP was <85mmHg. Rendering a minimum of 3 and a maximum of 6 measurements for each participant, we computed the average home BP.

*(2) Study blood pressure measurement*. To verify the home-measurement based preliminary classification of each medication-free participant as hypertensive or normotensive, trained personnel performed three additional BP measurements during the study session in a seated position after a 15-minute rest by means of sphygmomanometry (Omron M6; Omron-Healthcare-Europe B.V., Hoofdorp, Netherlands). We applied the regular World-Health-Organization/International-Society-of-Hypertension definition of hypertension and classified medication-free participants as hypertensive if their average study SBP was ≥140mmHg and/or their average study DBP was ≥90mmH (49). Medication-free participants were classified as normotensive if their average study SBP was <140mmHg and their average study DBP was <90mmHg. The final group assignment of medication-free participants was based on congruent home and study BP classification.

### 2.2 Design and procedure

In anticipation of the experimental session, all participants consumed a semi-standardized breakfast following written instructions and abstained from caffeine and alcohol consumption 24h prior to their arrival at the lab at 8:00h. Questionnaires were administered, and participants´ height and weight were measured. Participants received material and written instructions for saliva collection at home, before resting study BP was assessed.

To assess longitudinal changes in CHD risk factors, blood samples were collected at 11:30h, i.e., after a fasting for 3.5h since arrival. All participants were invited for identical blood sampling procedures scheduled after a minimum of 1.5yrs later (mean ± *SEM*=3.00 ± 0.06).

### 2.3 Cortisol sampling protocol

Study participants were asked to obtain saliva samples on two consecutive workdays using salivette collection devices (Sarstedt, Rommelsdorf, Germany). To assess the CAR, five saliva samples were collected immediately after awakening and 15, 30, 45, and 60min (S1-to-S5) thereafter. Further samples were taken at 16:00h and 22:00h (S6-to-S7). Participants were free to wake up in accordance with their normal schedule, but at the latest by 8:00h. They had to remain lying in bed for the first 15min, and to abstain from breakfast during the first 30min, i.e. until after collection of the +30min after awakening salivette. For the breakfast that followed, participants were asked to avoid coffee or juicy drinks. Moreover, participants were instructed not to brush their teeth during the first hour after awakening. They were also told to clean their mouth with water before each saliva collection. In addition, participants were instructed to complete a diary during the sampling period, assessing bed- and wake-up times as well as the accurate sampling times. In addition to self-reports, we used electronic monitoring devices (MEMS Track Cap, Aardex, Switzerland).

A total of 123 participants provided accurate cortisol samples for both consecutive sampling days, whereas 15 participants provided accurate cortisol samples but for two non-consecutive days. Furthermore, CAR data of 53 participants were accurate for only one sampling day because of incomplete (*n*=33) or inaccurate sampling (*n*=20) of the other day. See **Supplemental Material** for more details.

### 2.4 Biochemical analyses

#### 2.4.1 Cortisol

Participants were instructed to store their saliva samples in the refrigerator until sampling completion and to then send the collected samples to our laboratory as fast as possible. We stored saliva samples until study completion at –20°C. Biochemical analyses of cortisol [nmol/L] were performed with a competitive time-resolved fluorescence immunoassay (DELFIA) (50) in the Biochemical Laboratory of the University of Trier. Intra- (4.0-6.7%) and inter-assay (7.1-9.0%) coefficients of variation were ≤9.0%.

#### 2.4.2 Prospective CHD risk assessment

We assessed prospective CHD risk by measuring changes between baseline and follow-up assessment of the following biological risk factors: (1) the prothrombotic factors D-dimer [µg/L] and fibrinogen [g/L], the (2) pro-inflammatory measures interleukin (IL)-6 [pg/mL], tumor necrosis factor alpha (TNF-α) [pg/mL], and the acute phase protein C-reactive protein (CRP) [μg/mL], and (3) blood lipid profiles in terms of total cholesterol (tChol)/high-density lipoprotein cholesterol (HDL)-ratio. Fibrinogen and D-dimer were analyzed at the Center for Laboratory Medicine of the Bern University Hospital (Inselgruppe AG, Bern) applying standard quality procedures following the Clauss method (51) (fibrinogen) and a particle-enhanced immunoturbidimetric assay (INNOVANCE^®^ D-Dimer, Siemens Healthcare GmbH, Erlangen, Germany), respectively. Blood lipids were also analyzed in Bern using *in vitro* assays (enzymatic colorimetric, Roche, Mannheim, Germany). IL-6, TNF-α, and CRP were analyzed in the biochemical laboratory of the Biological Work and Health Psychology group at the University of Konstanz. Cytokines were determined with a high sensitivity chemiluminescence sandwich immunoassay (Meso Scale Discovery, Rockville, USA), while CRP was determined using a high-sensitive enzyme immunoassay (ELISA, IBL Hamburg, Germany). For more details see **Supplemental Methods**.

#### 2.4.3 HbA1c

HbA1c analyses were performed with *in vitro* assays for the quantitative determination of HbA1c IFCC [mmol/mol] in whole blood (Tina-quant^®^, Roche, Mannheim, Germany) (see **Supplemental Methods**).

### 2.5 Statistical analyses

Statistical analyses were performed using SPSS (Version26.0) statistical software packages for MacIntosh (IBM SPSS Statistics, Chicago IL, USA). All tests were two-tailed with level of significance set at *p*<.05. No outliers were excluded.

We *a priori* calculated power-analyses using G∗Power3.1. Following our previous findings, we expected an effect size of *f*=.35 with respect to group differences between HT and NT in CAR (6). Based on our hypotheses we expected comparably smaller differences between CHD-patients and HT. To allow to detect small effects of *f*=.10 in a 3(groups)-by-5(measurement points) repeated measurement ANOVA with a power of 90% and an observed average correlation of the repeated measures of *r*=.54 in cross-sectional analyses, the required total sample size is *N*=180.

A posteriori, we determined *f* from partial η^2^ (η^2^_p_) values using G*Power3.1. Effect size parameters *f* and *R* ^2^ changes are reported where appropriate (effect size conventions; small: *f*=.10,Δ*R* ^2^=.02; medium: *f*=.25,Δ*R* ^2^=.13; large: *f*=.40,Δ*R* ^2^=.26) *(* 52).

For all participants, we calculated mean arterial BP (MAP) based on the three BP study measurements by the formula MAP=(2/3*mean study DBP)+(1/3*mean study SBP) and body-mass-index (BMI) by the formula BMI=kg/m^2^.

For data and measures relating to cortisol, we calculated mean values of the two sampling days. To aggregate diurnal cortisol profiles for prospective analyses, we calculated mean total diurnal cortisol released during sampling days computed as area under the curve with respect to ground (AUC_dayCort_:S1-to-S7). Total CAR´s were calculated accordingly (AUC_CAR_:S1-to-S5) (53). Diurnal cortisol slopes were estimated following previous recommendations with one formula anchoring cortisol levels at awakening (Slope_Awake_) and the other anchoring the individual morning peak (Slope_Peak_) (54). Fibrinogen, D-dimer, tChol/HDL-ratio, IL-6, CRP, and TNF-α changes from baseline to follow-up assessment were computed by subtracting baseline values from follow-up values, with higher change values indicating increases in the respective parameters over time, i.e. over the follow-up period. Building on previous methods [e.g. (37, 55, 56)], we computed an aggregated coagulation index by averaging z-transformed change values of D-dimer and fibrinogen. For an aggregated inflammatory index, we accordingly averaged z-transformed change values of IL-6, CRP, and TNF-α.

**Figure 1:**
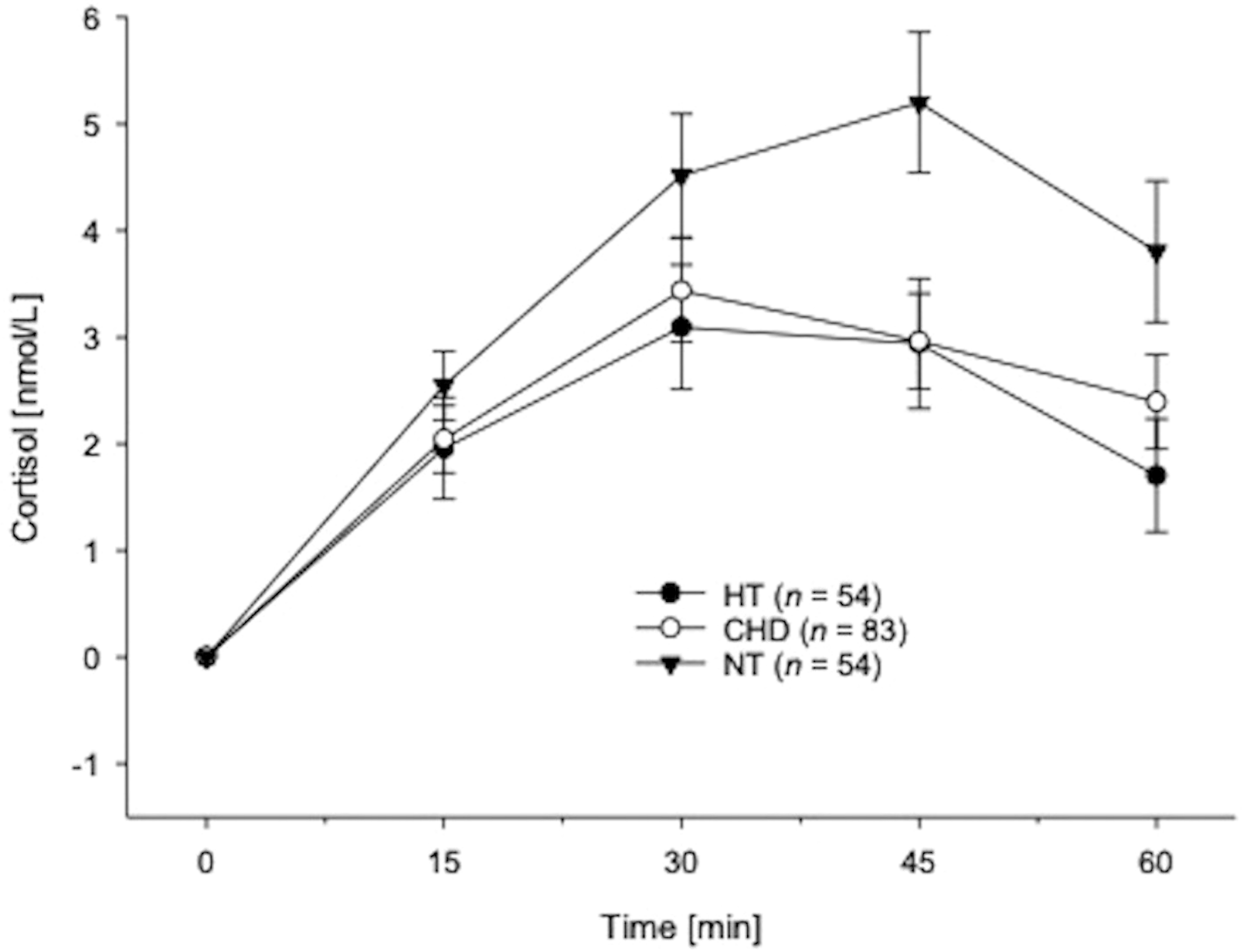
Cortisol awakening responses in hypertensive individuals, CHD-patients and normotensive participants without CVD calculated in terms of repeated measures ANOVAs and depicted as absolute changes from cortisol awakening concentrations (mean ± *SEM*).

**Figure 2:**
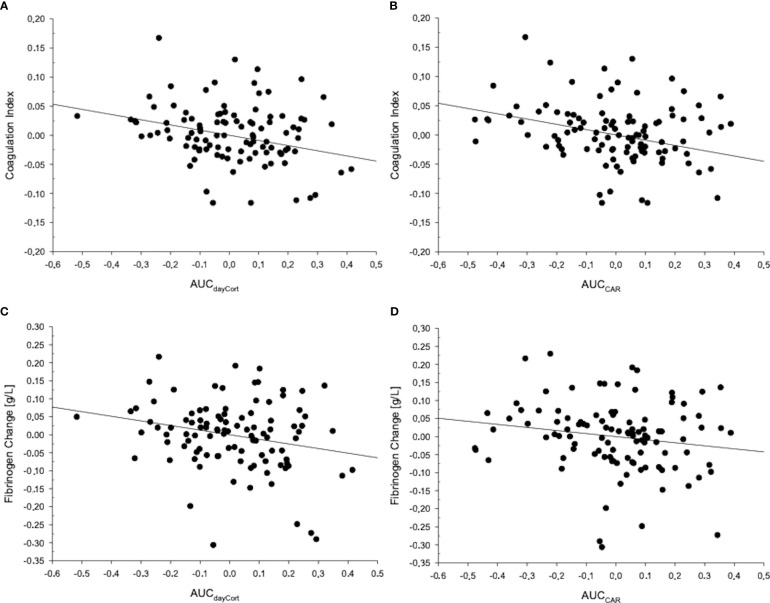
The figure depicts residuals of the respective dependent and independent variables adjusted for the full set of covariates. MANCOVA *post-hoc* testing, in terms of linear regression analyses, revealed that the coagulation index was significantly associated with **(A)** AUC_dayCort_ (*F*=7.55;*p*=.007) and **(B)** AUC_CAR_ (*F*=7.79;*p*=.006). Regression analyses revealed that fibrinogen change from baseline to follow-up was predicted by **(C)** AUC_dayCort_ (ß=-.26;*p*=.012) and **(D)** AUC_CAR_ (ß=-.17;*p*=.098).

All data were tested for normal distribution and homogeneity of variance using Kolmogorov-Smirnov and Levene’s tests prior to statistical analyses. All measures showing a skewed distribution (see **Supplemental Material**) were log-transformed. While log-transformed data were used for modeling and testing, we depict untransformed data in **Tables 1**, **2**, in **Figure 1** and in **Supplemental Tables S1, S2** for reasons of clarity. **Figure 2** depicts residuals of the dependent and independent variables adjusted for the full set of covariates.

**Table 2:** CHD risk factor changes between baseline and follow-up.

	Total *N*=106	CHD *n*=43	HT *n*=31	NT *n*=32
Time baseline to follow-up [months]	35.54 ± 0.77 (21–63)	33.70 ± 1.11 (21–58)	38.23 ± 1.03 (28–61)	35.41 ± 1.56 (21–63)
Coagulation				
Fibrinogen [g/L]	0.10 ± 0.05 (-1.10–2.22)	0.10 ± 0.07 (-0.77–1.27)	0.11 ± 0.07 (-0.78–0.97)	0.10 ± 0.11 (-1.10–2.22)
D-dimer [µg/L]	327.10 ± 51.48 (-3678–1575)	228.21 ± 100.78 (-3678–1102)	488.23 ± 61.87 (34–1575)	303.91 ± 80.04 (-1095–1288)
Inflammation				
IL-6 [pg/mL]	0.07 ± 0.04 (-1.14–1.41)	0.09 ± 0.05 (-0.93–1.41)	0.07 ± 0.05 (-0.48–1.00)	0.06 ± 0.08 (-1.14–1.27)
TNF-α [pg/mL]	0.11 ± 0.07 (-3.39–3.23) *n*=104	0.15 ± 0.09 (-1.04–3.23)	0.30 ± 0.09 (-0.36–2.31)	-0.15 ± 0.15 (-3.39–0.65) *n*=30
CRP [μg/mL]	0.41 ± 0.20 (-8.70–5.63) *n*=93	0.66 ± 0.25 (-2.11–5.63) *n*=40	0.15 ± 0.38 (-5.61–3.69) *n*=28	0.29 ± 0.46 (-8.70–4.04) *n*=25
tChol/HDL	0.18 ± 0.08 (-1.65–4.38)	0.15 ± 0.11 (-1.65–2.74)	0.09 ± 0.12 (-1.61–1.49)	0.30 ± 0.17 (-1.33–4.38)

To compute group differences in subject characteristics (**Table 1**) we used univariate ANOVAs. Cross-sectionally, to analyze whether groups differed in total diurnal cortisol secretion, we calculated repeated measures ANOVAs and ANCOVAs with repeated assessment of cortisol (S1–S7), as the dependent variable and group as the independent variable. *Post-hoc* testing comprised separate testing for differences between the 3 groups in the CAR (S1–S5), cortisol at awakening (S1), diurnal cortisol slopes (awake-to-last, peak-to-last), as well as evening cortisol (S7). We controlled for possible confounding effects of awakening time, sleep duration the night before saliva sampling, and medication intake in HT, in addition to age and BMI in repeated measures and univariate ANCOVAs (8). Further *post-hoc* testing comprised repetition of the previous cortisol analyses but with comparisons of two subject groups instead of three (i.e., HT-vs.-NT, CHD-vs.-NT, and CHD-vs.-HT). We applied Huynh–Feldt correction for repeated measures.

We calculated prospective analyses to shed light on the potential clinical relevance of basal HPA-axis activity. We tested whether diurnal cortisol parameters would predict future changes in CHD risk factors. We calculated multivariate analyses of covariance (MANCOVA) with prospective changes in blood lipids (tChol/HDL-ratio change), as well as the coagulation and inflammatory indexes as dependent variables. As the main independent variable of interest, we entered aggregated cortisol daytime levels (AUC_dayCort_). To avoid model overfitting given the reduced sample size of *N*=106 in our prospective analyses allowing for a maximum of 11 covariates simultaneously (57), covariates were entered setwise as follows: the default set of covariates comprises age at baseline, time between baseline and follow-up assessments, and medication intake in normotensive and hypertensive individuals at follow-up (model 1). Sleep duration and wake-up time (model 2), study group (model 3), and BMI at baseline in addition to prospective changes in BMI and MAP (model 4) were added successively as covariates to each previous model in complementary analyses (see **Table 3**). We *post-hoc* tested significant multivariate effects of aggregated cortisol daytime levels on future CHD risk by repeating the above described MANCOVA procedure while entering the separate parts of daytime cortisol secretion, i.e., CAR, diurnal slopes, and evening cortisol, as independent variable. *Post-hoc* testing of significant between-subject effects of cortisol parameters on any of the three dependent variables comprised linear regression analyses including changes where appropriate (e.g. D-dimer and fibrinogen change levels if the coagulation index was significant).

**Table 3:** Prediction of the prospective changes in CHD risk factors.

Cortisol	Model	Multivariate Tests		Tests of Between-Subjects Effects		
		*F*	*p*	Index	*F*	*p*
Main analysis	1	3.39	**.021**	Coagulation	7.56	**.007**
AUC_dayCort_				Inflammation	.44	.51
				tChol/HDL	.03	.85
	2	3.27	**.025**	Coagulation	8.27	**.005**
				Inflammation	0.10	.75
				tChol/HDL	0.009	.93
	3	3.22	**.026**	Coagulation	7.70	**.007**
				Inflammation	0.32	.57
				tChol/HDL	0.01	.92
	4	2.90	**.039**	Coagulation	7.55	**.007**
				Inflammation	0.11	.74
				tChol/HDL	0.04	.85
*Post-hoc*	1	3.17	**.028**	Coagulation	8.28	**.005**
AUC_CAR_				Inflammation	.09	.77
				tChol/HDL	.09	.77
	2	3.09	**.031**	Coagulation	8.30	**.005**
				Inflammation	0.04	.84
				tChol/HDL	0.09	.77
	3	2.98	**.035**	Coagulation	8.10	**.005**
				Inflammation	0.03	.87
				tChol/HDL	0.08	.78
	4	2.70	**.050**	Coagulation	7.79	**.006**
				Inflammation	0.01	.94
				tChol/HDL	0.20	.66
Waking cortisol	1	0.81	.49	Coagulation	2.17	.14
				Inflammation	0.80	.37
				tChol/HDL	0.14	.71
	2	0.83	.48	Coagulation	2.18	.14
				Inflammation	0.91	.34
				tChol/HDL	0.11	.74
	3	1.13	.34	Coagulation	2.75	.10
				Inflammation	1.46	.23
				tChol/HDL	0.01	.91
	4	1.04	.38	Coagulation	2.38	.13
				Inflammation	1.56	.22
				tChol/HDL	0.06	.80
Slope_Peak_	1	1.64	.18	Coagulation	4.70	**.032**
				Inflammation	0.01	.92
				tChol/HDL	0.004	.95
	2	1.62	.19	Coagulation	4.69	**.033**
				Inflammation	0.02	.90
				tChol/HDL	0.02	.90
	3	1.50	.22	Coagulation	4.39	**.039**
				Inflammation	0.02	.88
				tChol/HDL	0.04	.85
	4	1.25	.30	Coagulation	3.77	.06
				Inflammation	0.08	.78
				tChol/HDL	0.18	.67
Slope_Awake_	1	0.77	.51	Coagulation	1.67	.20
				Inflammation	0.99	.32
				tChol/HDL	.08	.78
	2	0.78	.51	Coagulation	1.64	.20
				Inflammation	1.01	.32
				tChol/HDL	0.10	.75
	3	1.24	.30	Coagulation	2.09	.15
				Inflammation	1.74	.19
				tChol/HDL	0.32	.57
	4	1.18	.32	Coagulation	1.82	.18
				Inflammation	1.97	.16
				tChol/HDL	0.19	.67
Evening cortisol	1	0.76	.52	Coagulation	2.17	.14
				Inflammation	0.002	.96
				tChol/HDL	0.13	.72
	2	0.76	.52	Coagulation	2.11	.15
				Inflammation	0.0001	.99
				tChol/HDL	0.15	.70
	3	0.80	.50	Coagulation	2.00	.16
				Inflammation	0.04	.85
				tChol/HDL	0.28	.60
	4	0.84	.48	Coagulation	1.90	.17
				Inflammation	0.15	.70
				tChol/HDL	0.18	.67

Covariates in model 1: age at baseline, time between baseline and follow-up assessment and medication at follow-up; model 2: additional control for sleep duration and wake-up time; model 3: additional control for study group; model 4: additional control for BMI at baseline as well as BMI and MAP changes between baseline and follow-up. Statistically significant results are highlighted in bold.

## 3 Results

### 3.1 Group characteristics

**Table 1** provides demographic and medical characteristics of CHD-patients, as well as hypertensive and normotensive participants. The three study groups differed in terms of age and BMI: CHD-patients had the highest average age (*p* ≤.001; mean ± *SEM*: CHD: 65.02 ± 0.99; HT: 52.74 ± 1.57; NT: 50.80 ± 1.63), whereas HT had a higher BMI than the other groups (*p*<.001; mean ± *SEM*: CHD: 27.85 ± 0.43; HT: 28.51 ± 0.52; NT: 25.27 ± 0.33). As expected, HT showed the highest study values in systolic BP, diastolic BP, and MAP compared with NT and CHD-patients (*p*<.001). HT showed the highest CRP (*p*<.004) and tChol/HDL-ratios (*p*<.001), while CHD-patients had highest fibrinogen and HbA1c levels (*p´s* ≤.010). On average, HT had serum levels of creatinine, calcium, sodium, and potassium in the normal reference range, thus supporting a diagnosis of essential hypertension. No participant had a diagnosis of a disease affecting the basal activity of the HPA-axis, such as adrenal insufficiency. In addition, all patients were under medication but no participant was treated with glucocorticoid substitution therapy (see **Supplemental Material**).

### 3.2 Diurnal HPA-axis activity

Repeated measures AN(C)OVAs with cortisol as repeated dependent variable (S1-S7) revealed that the three groups significantly differed in their total diurnal HPA-axis activity (interaction group-by-time: *F*(8.16,767.45)=2.73,*p*=.005,η^2^_p_=.03,*f*=0.18; with covariates: *F*(8.43,771.31)=1.95,*p*=.047,η^2^_p_=.02,*f*=0.14). As compared to normotensives, both HT and CHD-patients had lower cortisol concentrations during the day (HT-vs.-NT: interaction group-by-time: *F*(3.83,405.78)=2.85,*p*=.026,η^2^_p_=.03,*f*=0.18); with covariates: *F*(4.04,407.75)=3.31,*p*=.011,η^2^_p_=.03,*f*=0.18); CHD-vs.-NT: interaction group-by-time: *F*(4.13,556.91)=2.75,*p*=.026,η^2^_p_=.02,*f*=0.14; with covariates: *F*(4.25,557.01)=2.29,*p*=.055,η^2^_p_=.02,*f*=0.14). Moreover, CHD-patients had lower total diurnal HPA-axis activity as compared to HT, but not independent of covariates (interaction group-by-time: *F*(3.97,535.52)=2.63,*p*=.034,η^2^_p_=.02,*f*=0.14); with covariates: *p*=.54).

#### 3.2.1 Cortisol at awakening and cortisol awakening response

*Post-hoc* testing of total diurnal HPA-axis activity comprised further analysis of cortisol levels within the first hour after awakening (see **Figure 1**).

*Cortisol at awakening*. As depicted in **Table 1**, the three groups differed in their cortisol levels at awakening (*F*(2,188)=4.28,*p*=.015,η^2^_p_=.04,*f*=0.20; with covariates: *F*(2,183)=3.15,*p*=.045,η^2^_p_=.03,*f*=0.18). Cortisol awakening levels were highest in HT, in particular as compared to normotensives who showed lowest awakening levels (HT-vs.-NT: *F*(1,108)=7.85,*p*=.006,η^2^_p_=.07,*f*=0.27; with covariates: *F*(1,101)=8.38,*p*=.005,η^2^_p_=.08,*f*=0.29). Differences between HT and CHD-patients were of borderline significance (CHD-vs.-HT: *F*(1,137)=3.91,*p*=.050,η^2^_p_=.03,*f*=0.18; with covariates: *p=*.08). However, despite higher cortisol awakening levels, CHD-patients did not significantly differ from normotensives (CHD-vs.-NT: *p*=.18, with covariates: *p*=.20).

*Cortisol awakening response.* Repeated measures AN(C)OVAs with cortisol (S1-S5) as repeated dependent variable showed significant CAR group differences (interaction group-by-time: *F*(5.67,532.65)=3.10,*p*=.006,η^2^_p_=.03,*f*=0.18); with covariates: *F*(5.82, 532.19)=2.61,*p*=.018,η^2^_p_=.03,*f*=0.18). As compared to normotensives, HT and CHD-patients showed a lower CAR (HT-vs.-NT: interaction group-by-time: *F*(2.61,276.85)=4.89,*p*=.004,η^2^_p_=.04,*f*=0.20; with covariates: *F*(2.75,277.73)=4.59,*p*=.005,η^2^_p_=.04,*f*=0.20; CHD-vs.-NT: interaction group-by-time: *F*(2.70,365.06)=4.34,*p*=.007,η^2^_p_=.03,*f*=0.18; with covariates: *F*(2.80,366.70)=4.23,*p*=.007,η^2^_p_=.03,*f*=0.18). However, HT and CHD-patients did not differ in their CAR (*p*=.80; with covariates: *p*=.59).

#### 3.2.2 Diurnal decline and evening cortisol

The groups differed in terms of diurnal cortisol decline from awakening to evening, but not independently of covariates (Slope_Awake_: *F*(2,188)=3.48,*p*=.033,η^2^_p_=.04,*f*=0.20; with covariates: *p*=.11). Diurnal decline from awake to evening was steepest in HT who differed from normotensives with flattest awakening levels (HT-vs.-NT: *F*(1,106)=5.53,*p*=.021,η^2^_p_=.05,*f*=0.23; with covariates: *F*(1,101)=4.29,*p*=.041,η^2^_p_=.04,*f*=0.20). HT and CHD-patients in terms of diurnal decline but not independently of covariates (HT-vs.-CHD: *F*(1,135)=4.34,*p*=.039,η^2^_p_=.03,*f*=0.18: with covariates: *p*=.09), but CHD-patients did not significantly differ from normotensives (CHD-vs.-NT: *p*=.44; with covariates: *p*=.67).

In terms of evening cortisol, HT showed lower levels as compared to CHD-patients (HT-vs.-CHD:*p*=.08; with covariates: *p*=.79; HT-vs.-NT: *p*=.49; CHD-vs.-NT: *p*=.35; see **Table 1**).

### 3.3 Prediction of future CHD risk by diurnal HPA-axis activity

Our main MANCOVA analysis revealed that higher daytime cortisol levels in terms of AUC_dayCort_ significantly related to future overall CHD risk comprised the dependent variables of the MANCOVA: tChol/HDL-ratio change, coagulation and inflammatory indices (MANCOVA multivariate effects: model 1: *F*(3,99)=3.39,*p*=.021,η^2^_p_=.10,*f*=0.33,Wilk’sΛ=.91). Additional controlling for further covariates (models 2-to-4) did not alter the significance of this multivariate effect (*p* ´s≤.039, see **Table 3**). AUC_dayCort_ levels were significantly associated with the coagulation index (MANCOVA between-subject effects: model 1: *F*(1,101)=7.56,*p*=.007,η^2^_p_=.07;*f*=0.27; models 2-to-4: *p* ´s≤.007) but not with the inflammatory index (*p* ´s≥.51) or prospective changes in tChol/HDL-ratio (*p* ´s≥.85). Further analysis revealed that AUC_dayCort_ predicted greater increases from baseline to follow-up in fibrinogen (regression analyses: model 1: ß=-.23,*p*=.017,Δ*R* ^2^=0.12; model 2-to-4: ß ´s≥-.26,*p ´*s≤.012,Δ*R* ^2^≥.14) but not D-dimer (*p ´*s≥.23).

We tested *post-hoc* the significant multivariate effect of AUC_dayCort_ on future overall CHD risk. Cortisol awakening levels in terms of AUC_CAR_ significantly related to future overall CHD risk (MANCOVA multivariate effects: model 1: *F*(3,99)=3.17,*p*=.028,η^2^_p_=.09,*f*=0.31, WilksΛ=.91; model-2-to-4: *p* ´s≤.050) with AUC_CAR_ being associated with the coagulation index (MANCOVA between-subject effects: model 1: *F*(1,101)=8.28,*p*=.005,η^2^_p_=.08,*f*=0.29; models 2-to-4: *p* ´s≤.006) but not with the inflammatory index (*p* ´s≥.77) or prospective changes in tChol/HDL-ratio (*p* ´s≥.66). With respect to coagulation measures, AUC_CAR_ predicted greater increases from baseline to follow-up in fibrinogen (regression analyses: model 1: ß=-.17,*p*=.082,Δ*R* ^2^=.10; model 2-to-4: ß ´s≥-.17,*p ´*s≤.098,Δ*R* ^2^≥.10) and in D-dimer (regression analyses: model 1: ß=-.17,*p*=.076,Δ*R* ^2^=.06; model 2-to-4: ß ´s≥-.17,*p ´*s≤.094,Δ*R* ^2^≥.07) towards a trend for significance. Neither cortisol levels at awaking, slopes, nor evening cortisol levels were associated with future CHD risk (*p* ´s≥.18).

## 4 Discussion

The first objective of our study was to cross-sectionally compare diurnal HPA-axis activity between male CHD-patients, HT, and NT at baseline. The novelty of this study is the comparison between HT and CHD-patients. HT and CHD-patients showed lower *overall diurnal cortisol* saliva concentrations as compared to healthy controls with lowest concentrations in CHD-patients. We found a reduced *CAR* in HT and in CHD-patients as compared to NT corroborating previous findings (5, 6, 16, 17, 21). Moreover, HT and CHD-patients did not differ in their CAR. However, regarding *cortisol at awakening*, HT showed highest and NT lowest levels of the three study groups, with CHD-patients showing borderline significantly lower awakening levels compared to HT. These results are in line with previous research, with higher early morning salivary cortisol levels in unmedicated HT compared with healthy controls (15), whereas medicated HT showed lower early morning cortisol levels compared to NT (16). The latter points to a potentially normalizing effect of BP medication on cortisol levels at awakening. In line with this assumption, medicated CHD-patients did not significantly differ from normotensive individuals in their cortisol levels at awakening. Salivary waking or early morning cortisol was not associated with CHD-(measures) in most previous studies including heart patients (18–20). The increased morning cortisol levels in combination with a reduced cortisol response to awakening in our HT may indicate a generally altered HPA-axis activity in the early morning as observed in subjects suffering from a wide range of health problems (54, 58). Although, HT and CHD-patients did not differ from NT in their *evening cortisol* levels, patients had significantly higher levels as compared to HT. Evidence from other studies, however, points to cross-sectional associations between higher bedtime (20) or late night (15) cortisol levels in HT and CHD-symptoms. A potential reason for this discrepancy may be that we assessed cortisol at 22:00h, but not at bedtime or late night levels. Nevertheless, the higher evening levels in our patients may add to adverse cardiac outcomes as observed in other studies [e.g. (28)]. Diurnal cortisol *decline* from waking to evening (Slope_Wake_) was steepest in HT and flattest in NT, but not independent of covariates, driven by the comparably high awakening levels in HT. Diurnal decline from morning peak to evening (Slope_Peak_) did not differ between groups. Some previous studies pointed to an association between flatter cortisol slopes and greater cardiovascular risk (5, 24), so our results, with steeper, and thus more normative (59) cortisol declines in HT as compared to healthy controls, seem unexpected. However, in line with our findings, other studies could not detect any association between diurnal cortisol slope and CHD-measures (19, 25). We offer different explanations for these divergencies: First, the observed result of more normative slopes in hypertensive individuals was not independent of covariates. One of the covariates, later awakening time, was borderline significantly associated with steeper slopes in HT as compared to NT (*p*=.054). Second the formulas for the calculation of diurnal slopes differ between studies, rendering comparison of effects difficult (54). Since we calculated the slope using evening cortisol levels instead of levels at bedtime, the dynamics of diurnal HPA-axis activity may have been captured incompletely. Third, the observed group differences in diurnal slopes from waking to evening may to some extent be explained by the elevated morning cortisol levels of HT as compared to CHD-patients and normotensive controls. Taken together, we observed group differences in basal HPA-axis activity, with lower CAR and lower overall diurnal cortisol levels in CHD-patients and HT as compared to healthy controls.

We found evidence for aggregated daytime cortisol and CAR levels in predicting overall CHD risk (i.e. dependent variables in the MANCOVA). In detail, we found lower aggregated cortisol levels to predict higher increases in coagulation markers at follow-up, while inflammation markers and blood lipid profile were not associated with basal HPA-axis activity. Moreover, the prospective association between diurnal cortisol secretion and coagulation was mainly driven by the prediction of fibrinogen increases. So far, only few studies investigated associations between basal HPA-axis and prothrombotic activity: Evidence from cross-sectional studies points to an association between higher cortisol levels (36, 60) or dysregulated diurnal cortisol profiles on the one hand (37) and measures including prothrombotic activity on the other hand, which may explain why circulating cortisol had been associated with atherosclerotic vessel damage (60). We found a longitudinal association between lower diurnal HPA-axis activity and higher overall CHD risk increase comprising increases in all three aggregated biological risk factor indices, and in particular with fibrinogen increases. Despite evidence for cross-sectional associations between basal HPA-axis activity and inflammation (37) or hyperlipidemia (39, 61), we could not detect prospective associations between daytime cortisol or CAR levels and changes in inflammation markers and blood lipid profiles. These results are in line with a study in 9-to-10 year-old children where baseline cortisol did not predict blood lipid levels 1 year later (61).

The variability between the different measures of cortisol and the outcomes could possibly be attributed to the fact that single cortisol measures may only explain a small proportion of variance and are strongly influenced by situational effects [see (62)]. As a consequence, it requires repeated diurnal cortisol assessments to be able to detect associations with outcomes.

Our results suggest that dysregulation in terms of reduced CAR and lower overall daytime levels may represent an early indicator for increased cardiovascular risk. However, the clinical utility of the different measures of cortisol and the question about whether the monitoring of the HPA-axis activity facilitates the identification of high-risk individuals needs to be clarified in future studies. Further, the mechanisms underlying the observed HPA-axis activity dysregulation in hypertension and CHD are unclear. Since none of our participants had a diagnosis of adrenal insufficiency, we consider it unlikely that the observed lower HPA-axis activity in HT and patients result from adrenal insufficiency. One possible explanation may relate to (former) chronic stress experiences during disease development. Chronic stress has been proposed to play a role in both HT and CHD development that has been associated with altered diurnal HPA-axis activity (63, 64). According to the Allostatic-Load-Model chronic stress causes repeated activation of stress reactions including HPA-axis and sympathetic-adrenal-medullary axis reactivity which accumulate over time leading to compensatory stress system dysregulations in terms of allostatic load (65). Given the higher blood pressure and overall SNS activity in hypertension, that notably has been proposed to represent a potential consequence from allostatic load (63), the observed lower HPA-axis activity in HT may represent a compensatory allostatic load system dysregulation. Allostatic dysregulation can lead to allostatic overload with tissue and organ damage, including the cardiovascular, the immune, and the metabolic system (65). Future studies are needed to further elucidate the role of lifestyle factors (related to allostatic load) in diurnal HPA-axis activity of individuals with elevated CHD risk [see (14)] and whether our findings in salivary cortisol also apply to cumulative measures of cortisol output such as hair cortisol.

In our prospective analyses, we found that lower aggregated cortisol daytime levels and CAR predicted independent biological CHD risk factors and in particular prothrombotic activity about 3 years later. Studies investigating the effects of glucocorticoid excess [e.g. due to Cushing’s syndrome (66)] suggest effects on blood coagulation. However, no clear relationship between hypocortisolism and hypercoagulability has yet been established. It remains to be elucidated whether the observed prospective coagulation increases similarly represent a compensatory allostatic load system dysregulation resulting from the HPA-axis dysregulation (63, 65). Also, whether the observed lower CAR, either alone or combined with the higher prospective coagulation increases, relates to the higher occurrence of myocardial infarctions in the early morning hours (67) remains to be elucidated.

Limitations of our study include the relatively high drop-out rate and the wide follow-up range that we mainly attribute to logistic reasons. Participants who dropped out did not substantially differ in their characteristics from those completing the follow-up assessment, except for lower TNF-α and higher CRP levels at baseline (for both, see **Supplemental Material**). Also, apart from the CAR, we measured cortisol only twice and we assessed evening cortisol and not bedtime levels. Moreover, we cannot completely rule out potential effects of repeated thawing during transportation although salivary cortisol measurements have been shown to be quite robust against repeated freeze-and-thaw-cycles (8, 68). Moreover, the generalizability of our results is limited to middle-aged men of relatively high socioeconomic status and future studies are needed to further elucidate whether our findings also apply to women (19, 22) and participants with differing socioeconomic status (69). Also, recruitment *via* blood donor facilities may interfere with generalizability and we cannot rule out that the use of 24-hour automatic BP measurement would have been even more accurate to diagnose hypertension status compared to the applied two-step assessment procedure including repeated home and study BP measurement. Another limitation of our study relates to the medication of the CHD-patients. First, medication in general can affect salivary cortisol assessment at different levels (e.g., with effects on the composition of the saliva or direct effects on the cortisol synthesis) (70). Further, the effects of CHD-medication on the different parameters of diurnal cortisol secretion (e.g. CAR) have not been investigated systematically to the best of our knowledge (71), so the comparison between our medicated and unmedicated groups are to be interpreted with caution, as potential medication effects cannot be ruled out. Also, it is possible, that CHD-medication prevented substantial increases in CHD risk over time in our drug-treated participants. Finally, despite the prospective nature of our study we cannot draw definite conclusions regarding causality as we cannot exclude potential influences by other factors.

Strengths of our study comprise the use of MEMS caps combined with self-recording of sampling times allowing us to ensure the adherence to the study protocol. Further, we controlled for many potentially confounding variables including waking time and sleep duration and cortisol was assessed on two consecutive days (8).

In conclusion, we found evidence for a downregulation of HPA-axis activity in both, CHD and HT. Our results moreover suggest that lower diurnal HPA-axis activity seems to predict poorer cardiovascular health in HT and CHD by promoting a hypercoagulable state. A down-regulation of basal HPA-axis activity may therefore play a role in the pathogenesis and/or progression of atherosclerosis.

## Data availability statement

The raw data supporting the conclusions of this article will be made available by the authors, without undue reservation.

## Ethics statement

The studies involving human participants were reviewed and approved by Ethics committee of the Canton of Bern, Switzerland. The patients/participants provided their written informed consent to participate in this study.

## Author contributions

Conceptualization, PHW. Formal analysis, CD. Funding acquisition, PHW and RvK. Investigation, LT, CZ-H and RvK. Methodology, RvK, RW, HS, NM-B and PHW. Project administration, RvK and PHW. Supervision, RvK and PHW. Visualization, CD and PHW. Writing—original draft, CD and PHW. Writing—review and editing, RvK, RW, HS, NM-B and CZ-H. All authors contributed to the article and approved the submitted version.
