# Supplementary material for: Lower diurnal HPA-axis activity in male hypertensive and coronary heart disease patients predicts future CHD risk
Source: Front Endocrinol (Lausanne). 2023 Mar 10;14:1080938. doi: 10.3389/fendo.2023.1080938 (PMC10036761; doi:10.3389/fendo.2023.1080938)
Supplement: Supplementary file 1 [file DataSheet_1.pdf]

Supplementary Material

1 Supplemental Methods

Study participants

Exclusion criteria. Of a total of 219 participants who provided cortisol with sampling diary data, 12 had to be excluded because of white coat and masked hypertension and another 11 participants had to be excluded as they failed to comply with the cortisol sampling protocol. Furthermore, five participants had to be excluded from the cross-sectional and another two from the longitudinal analyses as they reported symptoms of acute infections on the study day.

Additional information on missing data

In two CHD-patients, information on MI, CABG, and LVEF≤ 40 % was missing. Due to technical problems sodium and potassium could not be analyzed in 7 and calcium could not be analyzed in 8 HT of the final study sample with 2 of them having a diagnosis of essential hypertension and being under antihypertensive medication. We measured HbA1c and tChol/HDL-ratio in all participants. Due to technical problems tChol/HDL-ratio was missing in 6 participants and HbA1c was missing in 7 participants. Baseline measurements for IL-6 and TNF-α were missing in three participants. CRP could not be determined in 23 participants at baseline and in 13 participants at follow-up (see Table 1-to-2). Reasons for missing data were problems with blood sampling or processing, and insufficient amount of sample for the respective analysis.

Cortisol sampling protocol

We considered cortisol data in our analyses only, if the participants adhered to the study protocol of the respective sampling day with a delay of less than 10min between awakening and S1, and of less than 75min between S1 and S5[1,2]. Eleven participants did not comply with the study protocol on both sampling days and had to be excluded. One participant, who completed only the first sampling day did not provide S6 and S7 samples and we replaced these missings by the S5 value.

1. Kudielka BM, Kirschbaum C. Awakening cortisol responses are influenced by health status and awakening time but not by menstrual cycle phase. *Psychoneuroendocrinology* (2003) 28(1):35–47.
2. Stalder T, Kirschbaum C, Kudielka BM, Adam EK, Pruessner JC, Wüst S, et al. Assessment of the cortisol awakening response: Expert consensus guidelines. *Psychoneuroendocrinology* (2016) 63:414–32.

Assays for the prospective CHD risk assessment and HbA1c

Prothrombotic Measures. Venous blood was drawn into polypropylene tubes containing 3.8% sodium citrate (Sarstedt, Nümbrecht, Germany). Citrate tubes were immediately centrifuged for 20min at 4°C at 2000g and plasma was pipetted into aliquots. D-dimer was analyzed using a particle-

enhanced immunoturbidimetric assay for the quantitative determination of D-dimers in human plasma (INNOVANCE® D-Dimer, Siemens Healthcare GmbH, Erlangen, Germany) on a Sysmex CS-5100 (Sysmex Europe, Norderstedt, Germany). Plasma fibrinogen levels were determined by a routine clotting assay applying standard quality procedures following the Clauss method. The intra- and inter-assay coefficients of variation were ≤7.9%.

Blood Lipids. We measured tChol and HDL from heparine-coated monovettes (Sarstedt monovette orange). Analyses were performed using in vitro assays (enzymatic colorimetric) for the quantitative determination of blood lipids in human plasma (Roche, Mannheim, Germany) on a Roche/Hitachi Cobas C Analyzer (Roche, Mannheim, Germany). Mean inter- and intra-assay CVs were ≤1.2% and ≤2.5% respectively.

Pro-inflammatory Measures. For the determination of IL-6, TNF-α, and CRP venous blood was drawn in EDTA-coated monovettes (Sarstedt, Nümbrecht, Germany), and immediately centrifuged for 10min at 2000g and 4°C. Obtained plasma was stored at -80°C until analysis. IL-6 and TNF-α levels were determined with a high sensitivity chemiluminescence sandwich immunoassay (Meso Scale Discovery (MSD), Rockville, USA). CRP was determined using a high-sensitive enzyme immunoassay (ELISA, IBL Hamburg, Germany). For IL-6, mean inter- and intra-assay CVs were ≤7.3% and ≤4.5%. For TNF-α inter- and intra-assay CVs were ≤10.1% and ≤3.4% respectively. For CRP mean intra- and inter-assay CVs were <5.1% and 6.1% respectively.

HbA1c. To measure HbA1c, venous blood was drawn into EDTA-coated monovettes, and analyses were performed with in vitro assays for the quantitative determination of HbA1c IFCC (mmol/mol) in whole blood (Tina-quant®, Roche, Mannheim, Germany) using Roche/Hitachi Cobas C Systems (Roche, Mannheim, Germany). Mean inter- and intra-assay CVs were ≤1.6% and ≤2.0% respectively.

Log-transformed data

All measures showing a skewed distribution (cross-sectional analyses: age, BMI, fibrinogen, D-dimer, tChol/HDL-ratio, IL-6, TNF-α, CRP, cortisol concentrations, $Slope_{Awake}$, mean awakening time, sleep duration, systolic blood pressure measured at home for screening and HbA1c; prospective analyses: AUC_{TC}, AUC_{CAR}, evening cortisol concentrations, mean awakening time, time between baseline and follow-up assessment, BMI change, tChol/HDL-ratio change, as well as inflammatory and coagulation indexes, D-dimer and fibrinogen changes) were log-transformed.

2 Supplemental Tables

Supplemental Table S1

Medication

[%]	CHD<br>*n* = 83	HT<br>*n* = 12
ACE inhibitors	32 (38.6)	4 (33.3)
AT2 antagonists	30 (36.1)	7 (58.3)
Anticoagulants	81 (97.6)	-
Alpha-blockers	3 (3.6)	1 (8.3)
Alpha-&beta-bl.	4 (4.8)	-
Beta-blockers	50 (60.2)	-
Antiarrhythmics	3 (3.6)	-
Lipid-lowering drugs	78 (94.0)	1 (8.3)
Antidiabetic medication	9 (10.8)	-
Insulin	1 (1.2)	-
Parkinson´s disease medication	1 (1.2)	-
Antidepressants	4 (4.8)	-

Supplemental Table S2

Comparison of baseline group characteristics between participants completing follow-up assessment and drop-out participants.

	Follow-up<br>(*n* = 106)	Drop-out<br>(*n* = 85)	*p*
Age [years]	57.49 ± 1.13 (28–80)	57.58 ± 1.47 (21–85)	.81
BMI [kg/m²]	27.03 ± 0.34 (19.78–38.90)	27.65 ± 0.44 (21.93–46.44)	.28
Study MAP [mmHg]	102.72 ± 1.12 (75.33–139.89)	102.89 ± 1.21 (79.83–132.00)	.92
Study SBP [mmHg]	140.09 ± 1.63 (109.33–189.67)	140.81 ± 1.65 (112.50–187.33)	.76
Study DBP [mmHg]	84.04 ± 1.11 (58.33–115.00)	83.93 ± 1.18 (62.67–108.00)	.95
Fibrinogen [g/L]	2.69 ± 0.05 (1.65–4.46)	2.75 ± 0.06 (1.47–4.25)	.50
D-Dimer [µg/L]	513.68 ± 52.03 (45–4765)	594.75 ± 74.90 (167–5177)	.11
IL-6 [pg/mL]	0.56 ± 0.04 (0.03–3.61)	0.54 ± 0.03 (0.09–1.57)	.93
		n = 82	
TNF-α [pg/mL]	2.10 ± 0.07 (0.80–4.91)	1.90 ± 0.07 (0.71–5.11)	.020
		n = 82	
CRP [µg/mL]	2.02 ± 0.19 (0.07–9.59)	2.76 ± 0.22 (0.35–11.55)	<.001
	n = 97	*n* = 71	
tChol/HDL	3.47 ± 0.09 (1.73–6.31)	3.56 ± 0.11 (1.76–6.41)	.60
		n = 79	
Cortisol at awakening [nmol/L]	4.42 ± 0.27 (0.02–14.83)	4.71 ± 0.32 (0.38–18.00)	.32
Cortisol at 16:00h [nmol/L]	1.79 ± 0.11 (0.14–6.51)	1.85 ± 0.12 (0.06–7.38)	.73
Cortisol at 22:00h [nmol/L]	0.77 ± 0.07 (0.04–6.10)	0.87 ± 0.08 (0.07–3.22)	.16
Cortisol $Slope_{Peak}$	-0.48 ± 0.03 (-1.63–0.18)	-0.48 ± 0.03 (-1.23–0.18)	.97
Cortisol $Slope_{Awake}$	-0.23 ± 0.02 (-0.87–0.17)	-0.25 ± 0.02 (-1.00–0.18)	.49
